# COVID-19 Teleconferencing Experiences of Caregivers & Spouses with Dementia from QA/QI Support Group Study

**DOI:** 10.1093/geroni/igab046.3582

**Published:** 2021-12-17

**Authors:** Roscoe Nicholson, Mary Mittelman, Maureen O'Connor, Andrew Nguyen, Rebecca Salant, Tiffany Donley, Cynthia Epstein-Smith, Steven Shirk

**Affiliations:** 1 University of Chicago, Evanston, Illinois, United States; 2 NYU Langone HealthNYU Grossman School of Medicine, NYU Grossman School of Medicine, New York, United States; 3 VA Bedford Healthcare System, Bedford, Massachusetts, United States; 4 Boston University School of Medicine, Boston, Massachusetts, United States; 5 NYU School of Medicine, NYU School of Medicine New York City, New York, United States; 6 NYU School of Medicine, New York, New York, United States; 7 NYU Langone Health, New York, New York, United States; 8 Bedford Veterans Administration Medical Center, Bedford, Massachusetts, United States

## Abstract

In summer 2020, researchers conducted a Quality Assurance and Quality Improvement (QA/QI) assessment of the NYU Langone Alzheimer’s Disease and Related Dementias Family Support Program’s adaptations in response to COVID by interviewing 10 participating spouse caregivers of persons with dementia (PWD). The primary adaptations were shifting from in-person to online services, changing support groups from biweekly to weekly, and offering an arts-based group for PWD daily rather than weekly. In the course of these interviews, all respondents described their adaptation to remote teleconferencing programming, and five also contrasted their experiences with those of the PWD. Methods: After transcription and de-identification, a codebook was created from the transcript content that included a priori topics of interest as well as emergent themes using framework analysis. These transcripts were then coded by two independent coders through an iterative process and consultation with the codebook creator, who also resolved any discrepancies between coders. Results: Respondents reported largely successful transitions to teleconferencing for themselves, though missing the physical contact afforded by meeting in-person. However, they also described some interactional challenges related to participants talking over one another, and suggested more active moderating to facilitate greater turn-taking. The respondents’ descriptions of the PWD’s response suggested a much less successful transition to teleconferencing. Challenges and barriers included lack of interest, difficulty following or participating in conversation, and teleconferencing creating confusion, such making it “hard for her to separate out when everybody's in the same place or not."

